# Use of Primary Prophylaxis with G-CSF in Acute Myeloid Leukemia Patients Undergoing Intensive Chemotherapy Does Not Affect Quality of Response

**DOI:** 10.3390/jcm14041254

**Published:** 2025-02-14

**Authors:** Valeria Mezzanotte, Giovangiacinto Paterno, Ilaria Cerroni, Lucrezia De Marchi, Kristian Taka, Elisa Buzzatti, Flavia Mallegni, Elisa Meddi, Federico Moretti, Francesco Buccisano, Luca Maurillo, Raffaele Palmieri, Carmelo Gurnari, Adriano Venditti, Maria Ilaria Del Principe

**Affiliations:** 1 Hematology, Department of Biomedicine and Prevention, University of Rome Tor Vergata, 00133 Rome, Italy; valeria.mezzanotte@ptvonline.it (V.M.); ilaria.cerroni@ptvonline.it (I.C.); lucrezia.demarchi@ptvonline.it (L.D.M.); kristian.taka@ptvonline.it (K.T.); elisa.buzzatti@ptvonline.it (E.B.); flavia.mallegni@ptvonline.it (F.M.); elisa.meddi@ptvonline.it (E.M.); frederico.moretti@ptvonline.it (F.M.); francesco.buccisano@ptvonline.it (F.B.); raffaele.palmieri@ptvonline.it (R.P.); carmelo.gurnari@ptvonline.it (C.G.); dlpmlr00@uniroma2.it (M.I.D.P.); 2 Hematology, Fondazione Policlinico Tor Vergata, 00133 Rome, Italy; luca.maurillo@ptvonline.it

**Keywords:** acute myeloid leukemia, granulocyte colony-stimulating factor, febrile neutropenia

## Abstract

**Background/Objectives**: The objective of our study was to evaluate the safety and efficacy of granulocyte colony-stimulating factor (G-CSF) as primary prophylaxis in adult patients with acute myeloid leukemia (AML) undergoing intensive chemotherapy. **Methods**: We retrospectively analyzed 112 AML patients treated with intensive chemotherapy at Fondazione Policlinico Tor Vergata in Rome between January 2014 and March 2024. Patients were divided into G-CSF and non-G-CSF (nG-CSF) groups. We assessed the incidence of neutropenia, its severity and duration; duration of hospitalization and its costs; incidence of febrile neutropenia (FN) and septic shock; duration of antibiotic therapy (ABT) and antifungal therapy (AFT); complete remission (CR) rates; measurable residual disease (MRD) status; relapse rates; and outcomes. **Results**: G-CSF administration significantly reduced the duration of neutropenia (median 14 vs. 18 days, *p* < 0.05) and length of hospitalization (median 28 vs. 35 days, *p* < 0.05), in both induction and consolidation therapy. There were no significant differences in CR rates (73% vs. 67%, *p* = 0.64), MRD negativity achievement (52% vs. 48%, *p* = 0.68), leukemia relapse rates (43% vs. 62%, *p* = 0.14), or overall survival (OS) (median 16.7 vs. 12.3 months, *p* = 0.3) between G-CSF and nG-CSF groups. Thanks to a shorter hospitalization, the use of G-CSF led to €300,000 in savings over the last 4 years. **Conclusions**: Our findings support the safety of G-CSF in AML patients, demonstrating no adverse impact on treatment response. G-CSF abbreviated the duration of neutropenia and hospitalization, highlighting its potential clinical and cost-effective role in AML treatment.

## 1. Introduction

Acute myeloid leukemia (AML) is an aggressive hematological malignancy characterized by the rapid proliferation of abnormal myeloid precursor cells in the bone marrow. It is the most common form of acute leukemia in adults, with an estimated 21,450 new cases and 10,920 deaths in the United States in 2019 [1]. The disease primarily affects older adults, with a median age of 68 years at diagnosis [2]. Despite advancements in therapeutic strategies, five-year survival rates remain around 30%, reflecting both disease complexity and treatment-associated morbidity and mortality [3]. The standard frontline treatment approach for AML typically involves intensive chemotherapy, often combining cytarabine with an anthracycline (known as the “7 + 3” regimen—seven days of cytarabine plus three days of daunorubicin or idarubicin). This regimen has been implemented with the administration of targeted agents such as FLT3 inhibitors or the anti-CD33 immunoconjugate monoclonal antibody (gemtuzumab ozogamicin) for specific subgroups [4,5]. In eligible patients, according to a risk-adapted strategy, standard chemotherapy may be followed by hematopoietic stem cell transplantation (HSCT). While this aggressive treatment approach aims to achieve remission and improve survival rates, it also carries a significant risk of treatment-related complications, primarily severe myelosuppression. This myelosuppression leads to a heightened vulnerability to infections, which can result in prolonged hospitalizations and increased morbidity and mortality among patients [6,7,8]. Recent therapeutic developments include chemo-free regimens combining hypomethylating agents (azacitidine or decitabine) with venetoclax (a BCL-2 inhibitor) or IDH1/2 inhibitors (ivosidenib or enasidenib), offering improved tolerability for older or medically frail patients [9,10,11]. Nevertheless, infections during treatment-induced cytopenia persist as a major clinical challenge across all therapeutic modalities. Indeed, the mortality rate associated with treatment-related toxicity and complications during induction therapy is estimated at 10% overall, rising to approximately 20–30% in patients over 60 years of age [8,12,13]. Efforts to mitigate these risks have included prophylactic antimicrobial strategies and supportive care measures.

Granulocyte colony-stimulating factor (G-CSF) has been widely used in oncology to manage chemotherapy-induced neutropenia. It is a glycoprotein that stimulates the generation, differentiation, and function of neutrophils [14]. In the context of AML, G-CSF has been studied as a potential supportive care measure to reduce the duration of neutropenia and the risk of infectious complications following intensive chemotherapy [15]. However, the use of G-CSF in AML remains controversial. While it has shown benefits in reducing the duration of neutropenia and antibiotic use, some in vitro studies have suggested that G-CSF might stimulate the proliferation of AML blasts. This concern arises from the fact that approximately 30–50% of AML cases express G-CSF receptors on their surface. However, the clinical relevance of this observation remains unclear [16,17]. This has led to conflicting results in clinical trials and a lack of consensus in treatment guidelines regarding its routine use in AML patients. Current guidelines, including those from the European LeukemiaNet (ELN) 2022 [2] and the National Comprehensive Cancer Network (NCCN) 2024 [18], do not recommend routine G-CSF use except in specific circumstances, such as for life-threatening infections.

Starting from 2019, given the progressive increase in the number of patients with AML referred to our center, in the attempt to reduce hospitalization and allow more efficient inpatient flow management, we began routine administration of G-CSF (Filgrastim) as primary prophylaxis for febrile neutropenia (FN). To evaluate the impact of this change on our clinical practice, we conducted a retrospective analysis with three main objectives:

(1) Assess the role of G-CSF given in induction by comparing complete remission (CR) rates, measurable residual disease (MRD) negativity/positivity after the first consolidation cycle, leukemia relapse rates, and overall survival (OS) rates between patients receiving or not receiving G-CSF;
(2) Analyze G-CSF’s effectiveness in decreasing infectious episodes by comparing the duration of neutropenia, FN and septic shock rates, and hospitalization length between both groups during induction and consolidation therapy;
(3) Evaluate the cost-effectiveness of the use of G-CSF.

Based on this, we aim to provide insights into the role of G-CSF in AML treatment and its potential benefits for patient outcomes and to address the ongoing controversy surrounding its use in this patient setting.

## 2. Materials and Methods

We conducted a single-center, retrospective observational study including 112 adult patients (>18 years) with newly diagnosed acute myeloid leukemia (AML) who underwent intensive chemotherapy. The study was carried out at the Hematology Department of Fondazione Policlinico Tor Vergata in Rome, Italy, between January 2014 and March 2024. The study adhered to the Declaration of Helsinki principles, and all patients provided written informed consent. Patients were stratified and treated following the ELN guidelines according to the version available at the moment of patients’ enrollment [2,19,20]. Starting from 2019, G-CSF (Filgrastim, 5 mcg/kg/day) was administered at least 48 h after the last chemotherapy infusion until one of the following criteria was achieved: absolute neutrophil count (ANC) was ≥1 × 10^9^/L for at least 3 consecutive days, ANC reached 10 × 10^9^/L in a single measurement, or treatment failure was documented (presence of circulating blasts in peripheral blood or >5% blasts in bone marrow). We analyzed patient data by dividing them into two groups: the G-CSF group, which received Filgrastim, and the non-G-CSF (nG-CSF) group. We evaluated the duration of neutropenia, duration of hospitalization, incidence of FN, septic shock, occurrence of other infectious complications, duration of intravenous antibiotic therapy (ABT) and antifungal therapy (AFT), rates of neutropenia recovery, CR rates, MRD negativity after the first consolidation, incidence of relapse, and OS. Neutropenia duration was defined as the interval from the first day of ANC below 1 × 10^9^/L after chemotherapy until recovery to ANC > 1 × 10^9^/L. Hospitalization length was measured from admission to discharge, with discharge criteria being the resolution of complications and ANC recovery or stabilization for patients with stable or progressive disease. FN was defined as fever (body temperature ≥ 38.3 °C or ≥38 °C for over one hour) during neutropenia, irrespective of microbiological positivity [21,22]. Sepsis and septic shock were defined and managed following the 2021 international guidelines of the “Surviving sepsis campaign” [23]. Other infectious complications were identified through clinical observation, microbiological analysis, or radiological imaging. The lengths of intravenous ABT and AFT were recorded from initiation to completion. CR and relapse definitions followed ELN classification standards. MRD assessment occurred post-first consolidation cycle, with quantitative Polymerase Chain Reaction (qPCR)-MRD negativity defined as per ELN 2022 guidelines [2] and Multicolour Flow Cytometry (MFC)-MRD negativity defined with a cut-off of 3.5 × 10^−4^ residual leukemia cells [24]. An economic evaluation was conducted to assess the potential cost savings associated with G-CSF use. The analysis considered the reduction in hospitalization duration and its associated costs, based on an estimated expense of €780 per patient per day of hospitalization.

### Statistical Analysis

Comparisons between groups were performed to assess differences in biological and clinical data using the Chi-squared test or Fisher’s exact test for categorical data and Mann–Whitney and *t*-tests in the case of continuous variables. All tests were 2-sided, accepting *p* < 0.05 as a statistically significant value. OS was calculated from diagnosis to last follow-up or death. Relapse-free survival (RFS) was defined as the time from the achievement of CR after induction therapy to disease relapse or death from any cause, whichever came first. Probabilities of OS and RFS were calculated using the Kaplan–Meier method. All statistical analyses were performed using SPSS v28.

## 3. Results

### 3.1. Patient and Treatment Characteristics

During the study period, 112 patients completed induction therapy, with 73 (65%) proceeding to consolidation. A total of 116 consolidation courses were administered. The median age at diagnosis was 57 years (range: 19–75). G-CSF primary prophylaxis was given to 31/112 patients (28%) during induction therapy and to 35/73 patients (48%) who received consolidation therapy, for a total of 116 consolidation courses. According to the 2022 revision of the ELN guidelines, patients were classified as follows: 21 patients (19%) were favorable, 57 patients (51%) were intermediate, 27 patients (24%) were adverse, and 7 patients (6%) were not categorizable due to incomplete genetic/cytogenetic data. Treatment regimens varied among patients: the most used regimen (40/112, 36%) was the “7 + 3” regimen, consisting of cytarabine (100 mg/m^2^/day via continuous infusion for 7 days) combined with daunorubicin (60 mg/m^2^/day for 3 days), in 17 patients in combination with midostaurin, and in 7 in combination with gemtuzumab ozogamicin. The daunorubicin/etoposide/cytarabine (ADE) regimen was administered to 25 patients, comprising cytarabine (100 mg/m^2^ twice daily for 10 days), daunorubicin (50 mg/m^2^ on days 1, 3, and 5), and etoposide (50 mg/m^2^ daily for 5 days). The FLA-IDA regimen was used in 14 patients and included fludarabine (30 mg/m^2^/day for days 1–5), followed by cytarabine (2 g/m^2^/day for days 1–5), and idarubicin (10 mg/m^2^/day for days 3–5). CPX-351, a liposomal formulation maintaining a fixed 5:1 molar ratio of cytarabine to daunorubicin, was administered to 12 patients on days 1, 3, and 5. Lastly, the mitoxantrone/cytarabine/etoposide (MICE) regimen was given to the remaining 19 patients, all aged 60 years or older. This regimen consisted of mitoxantrone (6 mg/m^2^/day), etoposide (80 mg/m^2^/day), and cytarabine (1 g/m^2^/day), administered over a period of 5 days. G-CSF primary prophylaxis was given to 31/112 patients (28%) during induction therapy and to 35/73 patients (48%) who received consolidation therapy. Table 1 summarizes the baseline characteristics of the G-CSF and nG-CSF groups.

### 3.2. Use of G-CSF in Induction and Consolidation Phases

During induction, patients in the G-CSF group experienced a significant shortening in the duration of neutropenia (median 14 vs. 18 days, *p* < 0.001) and length of hospitalization (median 28 vs. 35 days, *p* < 0.001). However, there were no significant differences in the incidence of FN (72% vs. 78%, *p* = 0.48), septic shock (10% vs. 12%, *p* = 0.74), or duration of intravenous ABT (median 12 vs. 14 days, *p* = 0.18), and AFT (median 10 vs. 11 days, *p* = 0.32).

A subgroup analysis was performed for patients receiving the “7 + 3” and FLA-IDA regimens. The results remained consistent with those observed in the overall population: in patients treated with “7 + 3”, G-CSF significantly reduced the duration of neutropenia (median 19 vs. 22 days, *p* < 0.001), hospitalization (median 36 vs. 41 days, *p* = 0.034), and ABT (median 15 vs. 20.5 days, *p* = 0.032). No significant differences were found in the incidence of FN (90% vs. 100%, *p* = 0.99) and septic shock (17% vs. 9%, *p* = 0.54). The duration of AFT could not be evaluated due to the limited number of patients receiving it in both groups.

In patients treated with the FLA-IDA regimen, G-CSF significantly reduced the duration of neutropenia (median 17 vs. 26 days, *p* = 0.019) and hospitalization (median 29 vs. 49 days, *p* = 0.011). All 14 patients in this subgroup experienced FN. No episodes of septic shock were reported in the G-CSF group, compared to two cases in the nG-CSF group. There were no significant differences in the duration of intravenous ABT (median 14 vs. 12 days, *p* = 0.687). AFT duration was again not evaluable due to the limited number of patients receiving it.

During consolidation courses (Table 2) the median duration of neutropenia (10 vs. 14 days, *p* < 0.001) and length of hospitalization (24 vs. 28 days, *p* = 0.04) were significantly abbreviated in the G-CSF group. Again, no significant differences were observed in FN incidence (73% vs. 80%, *p* = 0.388), septic shock incidence (6% vs. 8%, *p* = 0.731), and duration of ABT (median 8 days vs. 9 days, *p* = 0.322).

### 3.3. G-CSF Use, Response Outcome and Safety

The study found no significant differences between the G-CSF and nG-CSF groups in terms of treatment response and survival: CR rates were similar between the G-CSF and nG-CSF groups (73% vs. 67%, *p* = 0.64), and no significant difference in MRD status after the first consolidation cycle (52% vs. 48%, *p* = 0.68) was observed between the two groups. Also, the relapse rate (43% vs. 62%, *p* = 0.138) was not significantly different between the two groups.

**Figure 1:**
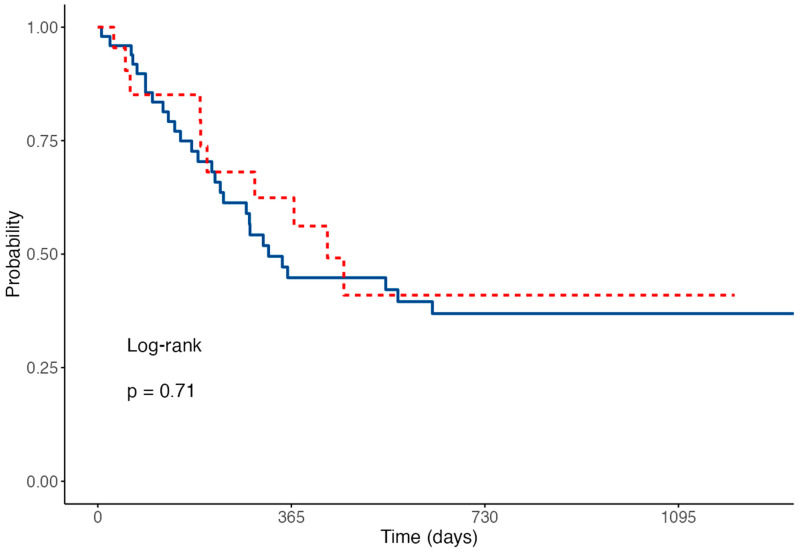
Kaplan–Meier overall survival curves for G-CSF (red) and nG-CSF groups (blue).

**Figure 2:**
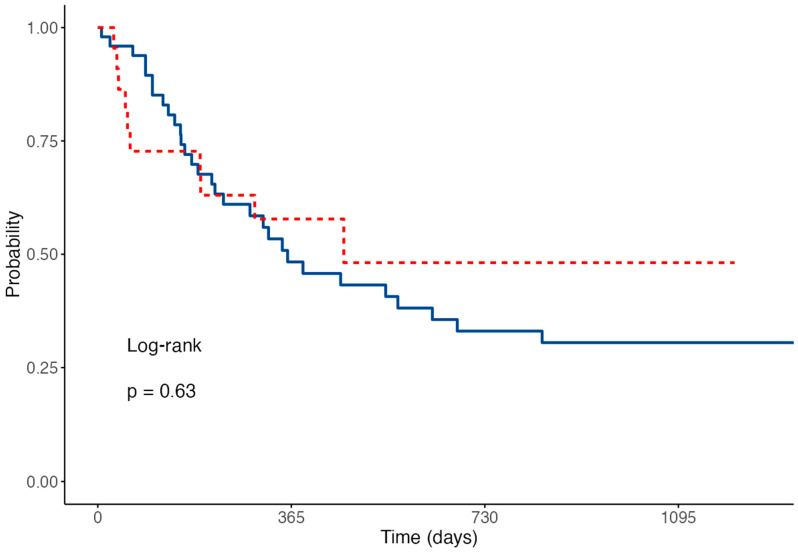
Kaplan–Meier relapse-free survival curves for G-CSF (red) and nG-CSF groups (blue).

OS and RFS were similar between the G-CSF and nG-CSF groups (median OS 16.7 vs. 12.3 months, *p* = 0.3, Figure 1; median RFS 15.4 months vs. 11.9 months, *p* = 0.63, Figure 2).

Subgroup analysis confirmed no significant differences in CR, OS, and RFS rates among patients treated with the “7 + 3” or FLAIDA regimens, whether receiving or not receiving G-CSF prophylaxis.

We then examined the potential economic impact of G-CSF use. Based on our internal sources, our department calculates an expense of €780 per patient per day of hospitalization. Therefore, the savings generated over the last 4 years were about €300,000. In general, we found that G-CSF administration was generally well tolerated. No patients experienced grade 3 or higher adverse effects related to G-CSF administration, as defined by the Common Terminology Criteria for Adverse Events (CTCAE), version 5 [25]. Only minor side effects were observed in a subset of patients. Specifically, 10 patients (32%) reported transient bone pain, headache, or skin rash. These side effects were generally mild and self-limiting, not requiring discontinuation of G-CSF or additional interventions.

## 4. Discussion

The use of G-CSF in AML patients undergoing intensive chemotherapy has been the subject of extensive study and debate, particularly in elderly patients. Our study contributes valuable insights to this ongoing discussion, particularly regarding the safety and efficacy of G-CSF as primary prophylaxis. Numerous randomized, controlled, prospective trials have evaluated G-CSF efficacy and safety in AML patients, yielding varied results. Furthermore, the findings of these studies and their implications for clinical decisions have often been deemed inconclusive. While it is well established that G-CSF can reduce the duration of neutropenia, its effects on CR rates and OS have been less clear, with some studies even raising concerns about potential negative effects on patient response and survival [15,16,26,27,28,29,30,31,32,33]. The European Organization for Research and Treatment of Cancer (EORTC)/Gruppo Italiano Malattie EMatologiche dell’Adulto (GIMEMA) AML-13 trial [29] investigated the impact of G-CSF on efficacy and toxicity when given in induction chemotherapy in elderly AML patients. This study, enrolling 722 patients, demonstrated that G-CSF administered concurrently with standard induction chemotherapy resulted in higher CR rates, shorter neutropenia durations, and reduced use of ABT and AFT. However, it showed no significant difference in infectious complications, mortality, or OS. Another phase 3 double-blind placebo-controlled study of Filgrastim in 521 de novo AML patients during induction and consolidation chemotherapy found that Filgrastim was effective and well tolerated, with no impact on CR or OS, even at a median follow-up of 7 years [31]. The study demonstrated significant reductions in fever duration, hospitalization, ABT, and AFT use. More recent studies have shown mixed results. A retrospective study conducted by Kang et al. reported reduced treatment-related mortality and FN duration with G-CSF use but no difference in OS or relapse rates [30]. Conversely, Wheatley et al. found similar CR rates but shorter OS in the G-CSF-treated patients, particularly in patients under 40 years old [33].

Several meta-analyses and reviews have attempted to shed some light on these conflicting results. Ottmann et al. reviewed 12 trials concluding that G-CSF is safe to use in both induction and consolidation therapy for AML patients, despite mixed results on CR and OS [34]. More recent meta-analyses from Bordbar et al. in pediatric patients showed no difference in relapse rate, OS, event-free survival, or mortality but suggested caution due to a higher relative risk of relapse in G-CSF-treated patients [35]. Similar considerations were proposed by Maeda et al., who suggested the selective use of G-CSF only in adult patients who are at a high risk of developing infectious complications during induction chemotherapy [17].

Despite the existence of large, randomized clinical trials demonstrating G-CSF safety, international guidelines still do not recommend its routine use. Many hematologists remain hesitant to use G-CSF as primary prophylaxis in induction, primarily due to its potential in stimulating blast outgrowth. This hesitancy is evidenced by a survey conducted by the “Sorveglianza Epidemiologica Infezioni Fungine nelle Emopatie Maligne” (SEIFEM) among 21 Italian hematology centers, which found that G-CSF was used as primary prophylaxis in only 14% of AML patients undergoing induction, a percentage significantly lower than in other hematological malignancies [36].

Our study adds additional insights into this ongoing debate. To our knowledge, this is the first time that G-CSF impact on disease outcome was analyzed via assessment of MRD status. Indeed, we demonstrated that G-CSF use did not alter the quality of response, with no significant differences in MRD negativity achievement and then relapse incidence. Similarly, CR rates and OS between G-CSF and nG-CSF groups were equivalent. At the same time, our results confirm that G-CSF is effective in reducing neutropenia duration and hospitalization length in both induction and consolidation regimens. We did not observe significant differences in septic shock rates or ABT/AFT duration, but this may be due to our limited sample size. A particularly critical aspect of our findings is the impact of G-CSF on the quality of life of patients undergoing intensive chemotherapy for AML. By shortening the duration of neutropenia and hospitalization, G-CSF not only facilitates quicker recovery but also allows patients to return to their daily lives sooner. The reduction in hospital stays is crucial for enhancing patients’ overall well-being during a challenging treatment process. Patients who spend less time hospitalized experience fewer disruptions to their personal lives, which can significantly improve their mental health and emotional resilience [37]. Furthermore, shorter hospital stays can alleviate the burden on caregivers who often face significant stress while managing their relatives’ health challenges. The ability to maintain more normalcy during treatment can lead to improved emotional support systems for patients, which are essential for coping with the psychological toll of cancer therapy [38].

The economic implications of G-CSF use in AML treatment are significant and often overlooked in clinical studies. Shortening hospitalization length has critical implications for healthcare systems, especially for those leveraging solidaristic taxation to provide national healthcare via Beveridge models. Indeed, the shorter duration of hospital stays during induction and consolidation and the lesser use of ABT/AFT associated with G-CSF administration led to estimated savings of around €313,000 in the last 4 years. Beyond its impact on direct medical costs, G-CSF use may contribute to broader economic benefits by facilitating earlier patient return to normal activities, thus reducing the burden on caregivers and improving quality of life. Finally, a shorter hospital stay is also desirable to reduce multidrug-resistant bacteria spread and minimize treatment program delays [39]. Our preliminary cost analysis suggested potential savings from reduced hospitalization; however, a formal cost-effectiveness model is needed. Our study has several limitations: the retrospective design introduces potential biases due to causal inferences; the relatively small sample size, particularly in the G-CSF group during induction therapy, and the heterogeneity of chemotherapy regimens may restrict the generalizability of our findings and the power to detect small differences in outcomes.

## 5. Conclusions

Our findings suggested that G-CSF does not negatively impact response or survival outcomes in AML patients. While not significantly altering clinical outcomes, G-CSF delivery abbreviates the duration of hospital admission and minimizes the occurrence of complications, therefore posing a compelling economic argument for its use, particularly in high-risk populations. The potential for cost savings makes the use of G-CSF a valuable add-on therapy in the management of AML. We believe that large, prospective studies are needed to provide more useful guidelines regulating its use in clinical practice.

## Figures and Tables

**Table 1:** Baseline patient characteristics of G-CSF and nG-CSF groups.

Patients Treated with Intensive Induction Chemotherapy 2014–2023 (n = 112)			
	G-CSF (n = 31)	nG-CSF (n = 81)	*p*-Value
**Sex (%)**			
Male	13 (41.93)	45 (55.56)	
Female	18 (58.06)	36 (44.43)	0.21
**Age at diagnosis, median (IQR)**	57 (13)	57 (20)	0.68
**WBC at diagnosis, ×10^9^/L, median (IQR)**	15.13 (26.2)	10.78 (43.18)	0.97
**PLTs at diagnosis, ×10^9^/L, median (IQR)**	98.00 (109.00)	51.00 (72.25)	0.06
**LDH at diagnosis, UI/L, median (IQR)**	452 (385)	414 (533)	0.92
**BMI > 30 (%)**	1 (3.23)	3 (3.7)	0.90
**2022 ELN risk group (%)**			
Favorable	5 (16.12)	16 (19.75)	ref.
Intermediate	14 (45.16)	43 (53.08)	0.94
Adverse	8 (25.8)	19 (23.45)	0.65
Not assessable	4 (12.9)	3 (3.7)	1
**CR, %**	73	67	0.64
**MRD negativity, %**	30	47	0.35
**Relapse, %**	43	62	0.138
**OS median, months (95% CI)**	16.7 (10.8–22.6)	12.3 (8.5–16.2)	0.3
**Induction chemotherapy regimen (%)**			
2 + 5	1 (3%)	0 (0)	
3 + 7 (alone) and 3 + 7-based regimens	11 (35%)	29 (36%)	
Fludarabine, idarubicin, cytarabine	9 (29%)	5 (6%)	
Fludarabine, cytarabine	0 (0)	1 (1%)	
Daunorubicin, cytarabine, etoposide	0 (0)	25 (31%)	
Mitoxantrone, etoposide, cytarabine	0 (0)	19 (23%)	
Lyposomial daunorubicin and cytarabine (CPX-351)	10 (32%)	2 (2%)	
**Neutropenia duration (days, median) (IQR)**	14 (5.5)	18 (10)	**<0.001**
**Febrile neutropenia, %**	72%	78%	0.48
**Septic shock**	10%	12%	0.74
**HD (days, median) (IQR)**	28 (10)	35 (13)	**<0.001**
**ABT duration (days, median) (IQR)**	12 (8.5)	14 (9)	0.18
**AFT duration (days, median) (IQR)**	10 (4)	11 (5)	0.32

WBC: white blood cell count; PLT: platelet count; LDH: lactate dehydrogenase; BMI: body mass index; G-CSF: granulocyte colony-stimulating factor; ELN: European Leukemia Net; CR: complete remission; MRD: measurable residual disease; OS: overall survival; HD: hospitalization duration; ABT: antibiotic therapy; AFT: antifungal therapy.

**Table 2:** Consolidation patients’ characteristics.

Consolidation Courses (n = 116)			
	G-CSF (n = 31)	nG-CSF (n = 81)	*p*-Value
**Consolidation chemotherapy regimen (%)**			
DIA	12 (24)	13 (20)	
High doses cytarabine	32 (64)	36 (55)	
Intermediate doses cytarabine	0 (0)	6 (9)	
Liposomial daunorubicin and cytarabine	4 (8)	0 (0)	
(CPX-351)			
Mitoxantrone, etoposide, cytarabine	1 (2)	0 (0)	
mini-ICE	1 (2)	11 (17)	
**Neutropenia duration (days, median) (IQR)**	10 (10)	14 (19)	**<0.001**
**Febrile neutropenia (%)**	73	80	0.48
**Septic shock**	6	8	0.74
**HD (days, median) (IQR)**	24 (8)	28 (6)	**<0.001**
**ABT duration (days, median) (IQR)**	8 (7)	9 (8)	0.18
**AFT duration (days, median) (IQR)**	10 (3)	12 (4)	0.32

DIA: daunorubicin + intermediate dose cytarabine; nini-ICE: mitoxantrone, etoposide, low-dose cytarabine; HD: hospitalization duration; ABT: antibiotic therapy; AFT: antifungal therapy.

## Data Availability

All data are presented in the paper and supplemental requests for additional information should be sent to the corresponding author.

## Abbreviations

The following abbreviations are used in this manuscript:

G-CSF	Granulocyte colony-stimulating factor
AML	Acute myeloid leukemia
FN	Febrile neutropenia
ABT	Antibiotic therapy
AFT	Antifungal therapy
CR	Complete remission
MRD	Measurable residual disease
ELN	European LeukemiaNet
NCCN	National Comprehensive Cancer Network
OS	Overall survival
ANC	absolute neutrophil count
RFS	Relapse-free survival
HD	Hospitalization duration
